# Is there a role of genetics in acute and chronic urticaria—A systematic review and meta‐analysis

**DOI:** 10.1002/clt2.70072

**Published:** 2025-07-09

**Authors:** George N. Konstantinou, Indrashis Podder, Arunima Dhabal

**Affiliations:** ^1^ Department of Allergy and Clinical Immunology 424 General Military Training Hospital Thessaloniki Greece; ^2^ Department of Dermatology College of Medicine and Sagore Dutta Hospital Kolkata West Bengal India; ^3^ Department of Dermatology Jagannath Gupta Institute of Medical Sciences and Hospital Kolkata West Bengal India

**Keywords:** gene polymorphisms, genetic polymorphisms, HLA‐B44, immune‐mediated disorders, vitamin D

## Abstract

**Background:**

Chronic urticaria (CU) is a heterogeneous skin disorder whose genetic drivers are incompletely defined.

**Objective:**

To systematically review and meta‐analyse genetic and epigenetic factors that influence susceptibility and treatment response in acute and CU.

**Methods:**

Following Preferred Reporting Items for Systematic Reviews and Meta‐Analyses guidelines, PubMed, Scopus and Web of Science were searched from inception to 31 July 2024. Original human studies reporting genetic or epigenetic associations with any urticaria subtype were eligible. Random‐effects meta‐analyses were undertaken when at least three comparable datasets were available.

**Results:**

Sixty‐one studies met the inclusion criteria. Associations were confirmed for HLA‐B44 (pooled Odds Ratio 8.15, 95% Confidence Interval 1.61–41.29; *I* ^2^ = 86%) and vitamin‐D‐receptor polymorphisms FokI, TaqI and BsmI, each conferring a 1.5‐ to 2.1‐fold increased risk. Variants in CRTH2, FcεR1α and C‐reactive protein predicted antihistamine response, while FCER1G, IL‐6, prostaglandin‐endoperoxide synthase 2, CCL2 and TNF‐pathway genes were over‐expressed in lesional tissue, supporting an immune‐mediated pathogenesis. Evidence for non‐CSU subtypes, acute urticaria, epigenetic modifications and gene–environment interactions was limited.

**Conclusions:**

CU displays an autoimmune‐like genetic signature. HLA‐B44 and vitamin D receptor variants are susceptibility markers, and pharmacogenetic signals enable personalised therapy. Longitudinal studies integrating environmental exposures and functional genomics are needed to translate these insights into precision care.

## 1 INTRODUCTION

Urticaria is a relatively common dermatological condition characterised by unpredictable onset of pruritic wheals with or without angioedema.^1^ Based on duration, it can be either acute (<6 weeks) or chronic (≥6 weeks). Acute urticaria (AU) affects approximately 20% of the global population at some point in their lives, while chronic urticaria (CU) affects about 1%.^2^ CU can either occur spontaneously (chronic spontaneous urticaria [CSU]), or be induced by specific triggers such as touch, pressure, or temperature variations (chronic inducible urticaria‐CIndU). CSU is the most common variant of CU, accounting for almost 80% of all cases.^3^ Despite being a non‐lethal and transient condition, urticaria, especially its chronic form, exerts a significant burden on both patients and the society.^4^

The pathogenesis of urticaria primarily involves the activation of mast cells and basophils. Recent understandings indicate two major pathways of activation in CU: IgE‐mediated autoallergy and IgG‐mediated autoimmunity. In the autoallergic pathway, IgE antibodies target autoantigens such as TPO and IL‐24, while in the autoimmune pathway, IgG autoantibodies are directed against the high‐affinity IgE receptor (FcεRI) or IgE itself. Degranulation of activated mast cells release several pro‐inflammatory mediators such as histamine, leukotriene and cytokines, subsequently resulting in the clinical manifestations of urticaria.^2^ Notably, both pathways may coexist in the same patient,^5^ and additional diverse pathogenetic mechanisms, such as direct degranulation of mast cells via the MRGPPX2 receptor or involvement of the coagulation cascade, have been implicated, as evidenced by the inadequate response to existing therapies in almost 40% patients.^6^

Emerging evidence indicates that almost one in every three CU patients may harbour an autoimmune disease, the most common being autoimmune thyroid disease.^7^

Genetic studies have begun to explore association with urticaria and polymorphisms in histamine‐related genes (e.g., FcεRI, HNMT), leukotriene‐related genes (e.g., ALOX5, LTC4S), the prostaglandin E2 receptor gene (PTGER4), and single nucleotide polymorphisms (SNPs) in pro‐inflammatory cytokine genes (e.g., IL‐6, TNF‐α).^8^, ^9^, ^10^ Recent genome‐wide association studies (GWAS) have shed more light on the genetic overlap between urticaria and other autoimmune diseases, suggesting a more robust genetic link with autoimmune conditions compared to atopic diseases.^10^ These associations suggests a potential shared genetic background between urticaria and autoimmune conditions in line with the increased prevalence of CU among first‐degree relatives.^11^ However, most of the information is sporadic in nature, and there is lack of systematic reviews in this context. A comprehensive understanding of the genetic factors involved in urticaria could elucidate its pathogenesis, predict therapeutic responses, and pave the way for precision medicine approaches tailored to individual patient profiles.

This systematic review seeks to bridge current knowledge on the genetic predispositions influencing the pathogenesis and management of urticaria. By exploring and analysing relevant genetic factors, it aims to highlight critical gaps and unmet needs in the field, establishing a foundation for further genetic research. Moreover, it will examine the existence of potential genetic‐based biomarkers to improve diagnostic accuracy and support the development of personalised therapeutic strategies to enhance outcomes for patients with urticaria.

## 2 METHODS

We conducted a systematic review following the Preferred Reporting Items for Systematic Reviews and Meta‐Analyses (PRISMA) guidelines. The study protocol was pre‐registered in the International Prospective Register of Systematic Reviews (PROSPERO) (no. CRD42024582623).

### 2.1 Main outcomes

The primary outcomes of the systematic review are:

- Genetic Associations with Urticaria: Identification of genetic polymorphisms, GWAS findings, and epigenetic modifications associated with acute or CU. Outcomes are defined by statistically significant associations (e.g., Odds Ratios [ORs], relative risk) between genetic markers in urticaria patients compared with controls (were available).
- Severity and Progression of Urticaria: The impact of genetic factors on severity and progression is measured by clinical scores such as the Urticaria Activity Score, which changes over time.
- Genetic Overlap with Other Autoimmune Conditions: Identification of genetic factors common to urticaria and other autoimmune/allergic conditions, measured by the frequency and significance of shared genetic markers.

### 2.2 Search strategy

A comprehensive search was undertaken across the PubMed, Scopus, and Web of Science databases to identify relevant studies published until 31 July 2024. Keywords and key phrases used for the research were: ‘chronic urticaria’ [MeSH Terms] OR ‘chronic spontaneous urticaria’ OR ‘chronic idiopathic urticaria’ OR ‘chronic inducible urticaria’ AND ‘genes’ OR ‘genetics’ OR ‘genome’ OR ‘polymorphism’. The complete search strategy of the three databases is included in the supplementary information. The reference lists of the screened articles were manually screened for further potentially eligible relevant publications (Box S1).

### 2.3 Article selection criteria

We used the PICO criteria to formulate the following criteria for this analysis:

#### 2.3.1 Inclusion criteria

- Population:
  - Studies involving human participants of any age/gender/ethnicity diagnosed with AU or CU, with or without angioedema,
  - Studies focussing on a population with a confirmed genetic component or susceptibility to urticaria,
- Intervention/Exposure: Studies investigating genetic factors, including but not limited to genetic polymorphisms, GWAS, epigenetic modifications, and familial inheritance patterns related to any type of urticaria.
- Outcome:
  - Studies reporting the association between genetic factors and incidence, severity, or progression of acute or CU,
  - Studies focussing on the genetic overlap between urticaria (with or without angioedema) and other autoimmune or allergic conditions.
- Type of articles: Only original research articles were eligible, including observational studies (cohort, case‐control, cross‐sectional), clinical trials, and case series with at least 2 cases, focussed on genetic aspects of urticaria.
- Language and Publication Date: Only English‐language articles were eligible, published in peer‐reviewed journals, up to 31 July 2024.

#### 2.3.2 Exclusion criteria

- Studies involving non‐human subjects (e.g., animal studies or in vitro research),
- Studies focussing solely on angioedema, even if it is the histaminergic type,
- Studies focussing on urticaria caused by drug(s) (e.g., NSAIDs), food(s), parasite(s), or contact urticaria, or urticaria as a secondary manifestation to other primary diseases (e.g., autoinflammatory syndromes, connective tissue diseases),
- Studies primarily concerning urticarial vasculitis,
- Narrative reviews, opinion pieces, editorials, letters to the editor, and conference abstracts without full text, non‐English language or non‐peer‐reviewed articles, and inability to obtain the full text formed our additional exclusion criteria.

### 2.4 Screening and abstraction process

Rayyan web software was used to assist in study screening and selection. Three researchers (GK, IP and AD) independently selected potentially eligible articles. The authors were contacted for clarification or to obtain missing data when necessary. The authors discussed and resolved any disagreements on the inclusion of the studies.

### 2.5 Data extraction and assessment of bias

Articles were screened by their titles and abstracts. The second stage consisted of reading the full texts of eligible articles independently by all three authors (GK, IP, and AD). The following data were extracted from each eligible study:

- Study characteristics: study design, sample size, study duration and location,
- Participant demographics: age, sex, ethnicity,
- Type of urticaria (spontaneous or inducible or both),
- Genetic data: polymorphisms, GWAS findings, epigenetic modification,
- Outcome measures: effect measures (OR, RR, HR) related to urticaria severity and progression.

### 2.6 Strategy for data synthesis

The data synthesis strategy involved both qualitative and quantitative methods tailored to the nature and quality of the available data. The primary goal was to integrate findings across studies to assess the genetic factors associated with acute and chronic urticaria. Data synthesis was conducted if at least three studies provided sufficient and comparable data on a specific genetic factor related to urticaria. A high level of consistency across studies was required for pooling data. Studies should have similar populations, interventions, and outcome measures. In cases where data were too heterogeneous, narrative synthesis was used to summarise and interpret the findings.

### 2.7 Statistical analysis

A meta‐analysis was conducted for any genetic factor with at least three studies providing sufficient and comparable data, as defined by the inclusion criteria. ORs and their corresponding 95% Confidence Intervals (CIs) were extracted from eligible studies. Log‐transformed ORs and standard errors were calculated for effect size estimation. A random‐effects model was applied using the restricted maximum likelihood method to account for potential between‐study heterogeneity. Heterogeneity was assessed using the *I* ^2^ statistic, with values of 25%, 50%, and 75% indicating low, moderate, and high heterogeneity, respectively, and Cochran's Q test to evaluate statistical significance. Meta‐analyses and visualisations, including forest plots, were performed using R software (version 4.4.2) with the meta and metafor packages, which offer robust tools for advanced meta‐analysis.^12^, ^13^, ^14^

### 2.8 Risk of bias assessment

The risk of bias (RoB) for the studies included in the meta‐analyses was assessed using the Newcastle‐Ottawa Scale (NOS), a widely used tool specifically designed for evaluating observational studies, such as case‐control and cohort designs.^15^ The NOS was selected because it provides a structured and comprehensive framework tailored to the unique challenges of non‐randomised research, unlike tools such as the Cochrane RoB tool, which are designed for randomised controlled trials. The NOS evaluates three key domains: selection of study groups, comparability of cases and controls, and the ascertainment of exposure or outcomes. Predefined criteria were applied to each domain: selection assessed whether the case definition was adequate, whether cases were representative of the target population, and whether controls were appropriately selected and clearly defined; comparability evaluated whether confounding variables, such as age and gender, were controlled through matching or statistical adjustments; and exposure examined the methods used to ascertain exposure (e.g., genotyping techniques), whether the same methods were applied to cases and controls, and whether non‐response rates were reported. The evaluation was performed independently by all authors based on these criteria, with any discrepancies resolved through discussion.

## 3 RESULTS

### 3.1 Systematic review

**FIGURE 1:**
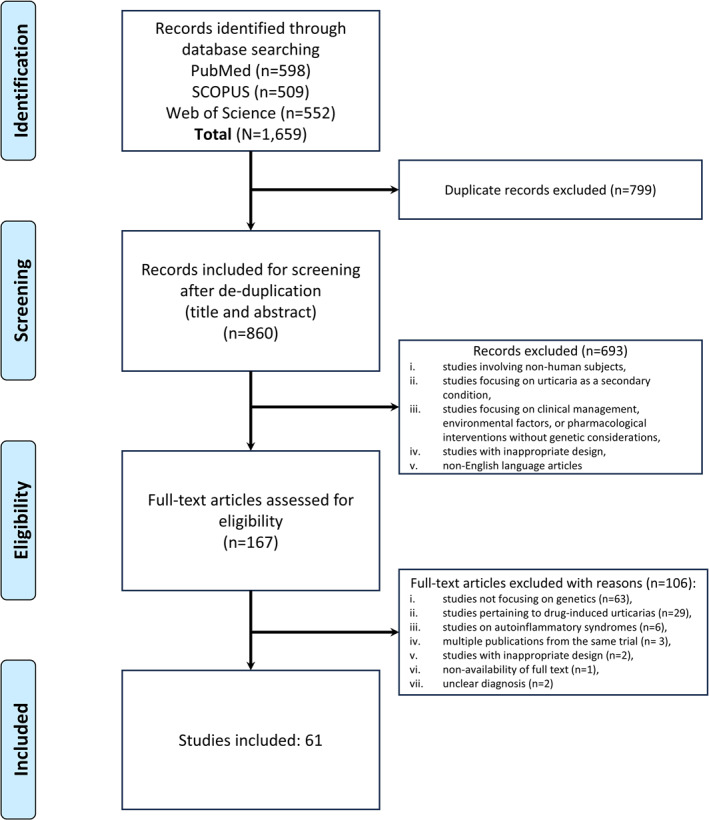
Preferred reporting items for systematic reviews and meta‐analyses (PRISMA) flow diagram of study selection.

Our literature search retrieved a total of 1659 articles of which 61 articles were eligible and included in the systematic review. The flow of articles has been depicted in the PRISMA flow‐diagram (Figure 1).

Among all the eligible articles (*n* = 61) (Table 1), the majority were cross‐sectional studies (*n* = 48), followed by record‐based genomic database studies (*n* = 5), Genome‐wide association study (GWAS) (*n* = 2), single‐group longitudinal trial (*n* = 2), one randomised, double‐blind placebo placebo‐controlled trial, one case‐control study and two case‐reports/series. Among the cross‐sectional studies, most included a control group (*n* = 46) while the remaining two studies were devoid of any control group.

**TABLE 1:** Details of included studies in the systematic review (*n* = 57).

First author, year	Place	Type of study/article	Sample size	Mean/median age (year) and gender of included CU patients	Genetic factors studied	Results	Remarks
Cohen, 1961^16^	United States	Case series	25 family members with chronic familial giant urticaria in a 5‐generation pedigree	NA	Familial occurrence	In five generations of 36 persons, there have been 25 with urticaria, and three deaths, two from laryngeal oedema, and one post‐surgically, from gastrointestinal oedema	The disease may be considered a distinct entity. It is inherited as a dominant trait, unresponsive to anti‐allergic medications and has a high mortality rate
Brachtel et al., 1979^17^	Germany	Cross‐sectional	239 patients with atopic conditions (61 patients with acute urticaria), 151 healthy controls	NA	The phenotype and gene distribution of 15 genetic blood polymorphisms (AB0, MNSs, Rhesus, P, Kell, Duffy, Kidd, Hp, Gc, Gm, Inv, aP, PGM∼, EsD, and 6‐PGD) were analysed and compared with those in 151 selected controls	Statistically significant distribution differences are only present regarding the polymorphic systems Kidd, Inv, and red cell acid phosphatase	Higher incidence of blood group antigens A and B in urticaria patients than in controls
Epstein et al., 1981^18^	USA	Single‐group cross‐sectional	28 relatives in a 6‐generation pedigree with dermo‐distortive urticaria	NA	Linkage analysis with 18 genetic markers (Transcobalamin II, PGM, ACP, GALT, GPT, HP, Gc, ABO, Le, Se, Rh, MNSs, FY, Kell, Kidd, P, PTC and HLA)	Close linkage of dermo‐distortive urticaria was not demonstrated with any marker	The linkage of dermo‐distortive urticaria with the MNSs blood antigen system is weakly suggested
						The most significant positive lod score was for the MNSs blood antigen system	
Gell et al., 1983^19^	USA	Cross‐sectional	1731 subjects with various disorders‐ dermatologic, rheumatologic, haematologic and others, (20 patients with urticaria and/or angioedema) 177 healthy controls	NA	Incidence of selective C4 deficiency in various diseases and any associated genetic mutation	Selective C4 deficiency was observed in 25% (5/20) patients with urticaria and/or angioedema, compared to 1.9% (34/1731) among all diseased patients	C4 deficiency is significantly associated with urticaria and/or angioedema
						The most common mutation associated with C4 deficiency was the genetic deficiency‐ Single null allele B*QO	
Pan et al., 1996^20^	Australia	Cross‐sectional	20 patients with CU, 20 healthy controls	Median age‐36 years Males‐8 Females‐12	Whether heterogeneity of the IgE (Ce) gene could be demonstrated in patients with chronic urticaria (CU) by DNA analysis from peripheral blood	Presence of a restriction fragment length polymorphism of the Ce gene segment in 20% (4/20) CU patients, but in none of 20 normal subjects	Polymorphism of the functional Ce gene may affect expression of this gene, possibly resulting in dysfunctional IgE‐receptor interaction with consequent alteration in mediator release
						Family studies of two propositi revealed the presence of this Ce gene polymorphism in other family members	
Lichtenstein et al., 1997^21^	Sweden	Cross‐sectional	1480 Swedish twin pairs, 7–9 years old	NA	The importance of genetic and environmental factors in asthma, hay fever, eczema, atid urticaria	Genetic/familial heritability for urticaria‐54% (boys), 60% (girls)	Although genetic factors are crucial in atopic diseases in children, both environmental and scx‐related factors are contributory
						Impact of environmental effects‐21% (boys), 25% (girls)	
			258 patients with urticaria, type not specified			Impact of non‐shared environmental effects (e.g., gender)‐25% (boys), 15% (girls)	
O'Donnell et al., 1999^22^	United Kingdom	Cross‐sectional	100 Caucasian patients with CIU 603 healthy controls	Mean age 41 ± 13.9 years Males‐52 Females‐48	Link between major histocompatibility complex (MHC) class II alleles and CIU, and compare with healthy controls	HLA DRB1*04 (DR4) and its associated allele, DQB1*0302 (DQ8), are significantly raised in CIU patients, compared to healthy controls (*p* = 0.0002)	There is a strong link between HLA class II alleles and CIU, supporting its autoimmune pathogenesis
						HLA DRB1*15 (DR15) and its associated allele, DQB1*06 (DQ6), are significantly reduced in CIU patients (vs. controls, *p* = 0.000036)	
						The HLA DRB1*04 association is stronger in ASST+ CIU patients, compared to those with negative ASST	
Oztas et al., 2004^23^	Turkey	Cross‐sectional	42 Turkish patients with chronic urticaria,	Mean age‐40.4 ± 12 years Males‐10 Females‐32	Association between human leucocyte antigen (HLA) class II alleles [HLA‐DR and HLA‐DQ] and chronic urticaria	The frequency of HLA‐DR4 was significantly higher in CU patients, compared to healthy controls (*p* = 0.01)	HLA class II alleles may be involved in the pathogenesis of CU, and may trigger the activation of immune system
			115 healthy controls			No other allele distribution was significantly different	
Chen et al., 2005^24^	China	Cross‐sectional	42 patients with CU, 193 age‐sex‐race matched healthy controls	Mean age 30.67 ± 12.45 years, Males‐19 Female‐23	Association of genotypes of HLA‐DRB1 and HLA‐DQB1 alleles with the genetic susceptibility of chronic urticaria (CU)	Gene frequencies of HLA‐DRB1*12, *0901 (RR = 3.11, chi2 = 7.579, *p* = 0.006; RR = 2.47, chi2 = 5.684, *p* = 0.017) were significantly increased in CU patients (vs. controls) Gene frequencies of HLA‐DQB1*05 (RR = 0.26, chi2 = 6.683, *p* = 0.01) were significantly decreased in CU patients	HLA‐DRB1*12, *0901 → possible markers for genetic susceptibility to CU HLA‐DQB1*05 → possible resistive role for CU
Pacor et al., 2006^25^	Italy	Cross‐sectional	69 patients with aspirin‐intolerant CU, and 200 healthy subjects	NA	Association between major histocompatibility complex class I and II alleles (HLA‐ A, B, Cw and DRB1) and aspirin‐intolerant CU	On multiple logistic regression analysis, HLA‐Cw4 and HLA‐Cw7 were associated with lower risk of aspirin‐intolerant CU, whereas HLA‐B44 was associated with a higher risk	There is possible association between some HLA class‐I antigens and aspirin‐intolerant CU
Aydogan et al., 2006^26^	Turkey	Cross‐sectional	55 CSU patients and 104 genetically unrelated controls	Mean age‐37 years Males‐16 Females‐39	Association between major histocompatibility complex class I and II alleles (HLA‐A, B, Cw, DR, DQ) and CSU	HLA Bw4 and DQ1 were significantly higher in CSU patients, while HLA‐A24 was significantly higher in controls	HLA‐Bw4 and DQ1 antigens may be associated with CSU susceptibility, while HLA‐A24 may have a protective role
Hosseini et al., 2006^27^	Iran	Cross‐sectional	40 patients with CU and 41 healthy controls	Mean age‐32 years Males‐8 Females‐32	To evaluate any relationship between the polymorphism of TGF‐β1 gene promoter (−509C>T) and chronic idiopathic urticaria	Type of polymorphism detected in CU patients‐ CC (11[27.5%]), CT (26 [65%]) and TT (3[7.5%])	Genetic variability at the promoter of TGF‐β1 gene (−509C>T) may increase the susceptibility to CU
Akcali et al., 2008^28^	Turkey	Cross‐sectional	95 CSU patients and 161 healthy subjects	Mean age‐38 years Males‐32 Females‐63	Association between angiotensin‐converting enzyme (ACE) insertion/deletion (I/D) polymorphism and CSU	No significant difference was found in allele frequencies of ACE I/D between the groups, but frequency of I allele was significantly higher in CSU patients with accompanying angio‐oedema compared to those without angio‐oedema. This group also has an over‐representation of II genotype	No evidence of association found between ACE I/D polymorphism and risk of CSU, but it may be involved in the molecular pathogenesis of angio‐oedema in CSU
Coban et al., 2008^29^	Turkey	Cross‐sectional	40 CU patients and 30 healthy controls	Mean age‐34.09 ± 7.25 Males‐14 Females‐26	Association between major histocompatibility complex class I and II alleles (HLA‐A, B and DRB1) and CU	Significantly higher frequency of HLA‐B44, HLA‐DRB1*01 and HLA‐DRB1*15 alleles was noted in the patients as compared to controls	Positive association of HLA‐B44, HLA‐DRB1*01 and HLA‐DRB1*15 alleles with CU suggests a genetic component in its pathogenesis
Palikhe et al., 2009^30^	South Korea	Cross‐sectional	384 patients with CU and 231 healthy controls	Mean age‐ NA Males‐173 Females‐211	Association of two CRTH2 gene polymorphism (−466T>C and −129C>A) with the CU phenotype and antihistamine drug requirement	Both genetic polymorphisms were statistically comparable between CU patients and healthy controls No association of genetic polymorphisms with disease characteristics viz. duration, atopy, serum total IgE CU patients with homozygous TT genotypes had significantly higher dose requirements of antihistamines (164.56 ± 115.62 vs. 137.38 ± 90.15 loratadine equivalents, mg/week) than those with the CT and CC genotypes (*p* = 0.025)	CRTH2 gene polymorphism (TT > CT/CC) may be associated with antihistamine response Limited evidence of association with disease characteristics
Bozek et al., 2010^31^	Poland	Cross‐sectional	115 patients with CSU, 162 healthy subjects	Mean age‐35.07 ± 6.21 years Males‐58 Females‐57	To investigate the HLA status of Polish patients diagnosed with CSU	HLA type significantly more in CSU patients → B44, DRB1*04 HLA type significantly more in healthy controls → A33 HLA‐C alleles and HLA‐DQ did not differ significantly between the groups	HLA alleles may be involved in CSU development or play a protective role in CSU
Di Lorenzo et al., 2011^32^	Italy	Cross‐sectional	94 CSU patients and 109 controls	Mean age‐39 years Males‐24 Females‐70	Five candidate polymorphisms of cyclooxygenases (COX) 1 and 2, and of 5‐lipo‐oxygenase‐activating protein (FLAP) were compared between cases and controls	COX‐2 5′UTR T/G, COX‐2 Exon 10 T/C, and FLAP‐336 G/A polymorphisms were significantly associated with both aspirin‐tolerant and aspirin exacerbated CSU	Polymorphisms of genes involved in prostaglandin and leukotriene metabolisms may be associated with CSU risk
Brzoza et al., 2012^33^	Poland	Cross‐sectional	91 CU patients, all ASST+ (autoimmune CU) 100 healthy controls	Mean age‐38.1 years Males‐27 Females‐64	To evaluate whether polymorphisms of the PTPN22 gene increase the risk for developing CU	Higher prevalence of −1123 C allele among CU patients 3 SNPs of PTPN22 gene significantly associated with CU → rs2488457C, rs1310182T and rs3811021T	PTPN22 gene polymorphisms may be associated with susceptibility for autoimmune CU
Zhou et al., 2012^34^	China	Cross‐sectional	220 patients with allergic skin disorders (including 123 CU patients), 286 children with asthma, and control groups of 283 adults and 208 children	NA	SNPs (rs2427837 A/G and rs61828219T/G) in the promoter region upstream of FCER1A exon 1	There was no difference in genotype distribution of either SNP between CU patients and controls	There was no significant risk of CU associated with the SNPs studied, although rs2427837A/G polymorphism increased the risk of development of AD
Brzoza et al., 2012^35^	Poland	Cross‐sectional	93 ASST‐positive patients with CU 105 healthy controls	Mean age‐38.5 years Males‐28 Females‐65	To investigate whether programed cell death 1 gene (PDCD1) polymorphisms influence susceptibility to CU PDCD1 gene plays an important role in pathogenesis of several autoreactive disorders	No statistically significant differences were found between CU patients and controls for allele or genotype distribution (PD1.3 and PD1.5 polymorphisms) No association between PDCD1 genotypes and severity of urticaria or age of disease onset	PDCD1 polymorphisms do not influence the susceptibility for autoimmune CU
Calamita et al., 2012^36^	Brazil	Cross‐sectional	42 CSU patients with positive ASST and 1000 genetically unrelated voluntary blood donors	Mean age‐44 years Males‐14 Females‐28	Association between major histocompatibility complex class I and II alleles (HLA‐ A, B and DRB1), and ASST‐positive CSU	No statistically significant difference was found in the prevalence of the HLA alleles studied between the two groups	No specific HLA association was found with CSU
Kai et al., 2013^37^	United Kingdom	Case‐report	Twin brothers with aquagenic urticaria	18 years, male	To explore any genetic component of aquagenic urticaria	Aquagenic urticaria in monozygotic twins, proven by water provocation test	Concurrence in monozygotic twins suggest a genetic background, but no specific genetic mutation identified
Tavakol et al., 2014^38^	Iran	Cross‐sectional	90 patients with CIU 140 healthy controls	NA	To examine the possible association of polymorphisms of TGF‐β and IL‐10 genes with susceptibility to CIU Polymerase chain reaction (PCR) was done to determine the genotype at 5 polymorphic sites; TGF‐β (codon10C/T and codon25G/C) and IL‐10 (−1082G/A, −819C/T, and −592C/A)	The C allele at codon 25 of TGF‐β was more prevalent in CIU patients compared to controls (*p* < 0.001) Genotypes of CT and CG at 10 and 25 codons of TGF‐β gene, respectively, and AG, CT, and CA for loci of −1082, −819, and −592 of IL‐10 gene were significantly higher in CIU patients (*p* < 0.001) In haplotype analysis, frequency of TGF‐β haplotypes differed between patients with CIU and controls; CC haplotype was overrepresented, while CG and TG haplotypes were underrepresented (*p* < 0.001)	TGF‐β and IL‐10 genetic variability could contribute to susceptibility to CIU Patients with CIU seem to have genotypes resulting in high production of TGF‐β and IL‐10
Brzoza et al., 2014^39^	Poland	Cross‐sectional	128 patients with autoreactive CSU (ASST+) 101 healthy volunteers	Mean age 38.8 years Males‐41 Females‐87	To test the possible role of the CTLA4 gene in the susceptibility to CU In all examined subjects CTLA‐4 A49G polymorphism was analysed	No statistically significant differences in the allele or genotype distribution between urticaria patients and controls were observed No association detected between CTLA4 polymorphism and urticaria severity or age of disease onset	There is no contribution of CTLA‐4 A49G polymorphism to chronic spontaneous autoreactive urticaria susceptibility
Brzoza et al., 2014^40^	Poland	Cross‐sectional	153 CSU patients, all ASST+ 115 healthy volunteers	Mean age‐38.2 years Males‐40 Females‐113	To evaluate whether CCR2 and CCR5 polymorphisms influence the susceptibility to CU in the Polish population CCR2 G190A and CCR5 d32 polymorphisms were studied in all subjects	Prevalence of CCR2 A allele was 1.7 times higher in the CU group, compared to healthy volunteers (*p* = 0.07) 1.8 times lower frequency of CCR5 d32 allele in the CU group, compared to controls (*p* = 0.05)	There may be possible role of CCR2 and CCR5 genes in the pathogenesis of autoimmune CU
Tavakol et al., 2014^9^	Iran	Cross‐sectional	90 patients with CIU 139 healthy controls	Mean age‐NA Males‐15 Females‐75	To evaluate any role of IL‐6 and TNF‐α gene polymorphisms in CIU susceptibility IL‐6 (G/C −174, G/A nt565) and TNF‐(G/A −308, G/A −238) polymorphisms evaluated in every subject	Genetic polymorphisms significantly more common in CIU than controls‐GG at −308 and −238 of TNF‐α gene; GG at −174 and GA at +565 of IL‐6 gene Genetic polymorphisms significantly less common in CIU‐AG at −308 and GA at −238 of TNF‐α gene, CG at −174 and GG at +565 of IL‐6 gene	Both TNF and IL‐6 genes may play some role in the pathogenesis of CIU
Guo et al., 2015^41^	China	Cross‐sectional	191 CSU patients 177 healthy controls	Mean age‐36.2 years Males‐77 Females‐114	To investigate whether FCER1A polymorphisms are associated with the risk of CSU, and to determine whether these polymorphisms influence the therapeutic efficacy of nonsedating H1‐antihistamines	There was significant difference in the allele frequency of rs2298805A between CSU patients and 177 healthy subjects (5.3 vs. 10.2%, *p* = 0.012, OR = 0.491, 95% CI 0.278–0.865) Significant difference in the allele frequency of rs2298805A between effective and ineffective groups (7.5 vs. 1.0%, *p* = 0.015, OR = 8.328, 95% CI 1.1–63.039) No difference in other alleles (rs10908703 and rs2494262) either between CSU patients and healthy controls, or between effective and ineffective groups	FCER1A rs2298805 genotype may increase the risk for CSU and influence therapeutic efficacy of nonsedating H1‐antihistamines in Chinese patients with CSU
Rasool et al., 2015^42^	India	Cross‐sectional	120 CSU patients 120 healthy controls	Mean age‐28.6 Males‐10 Females‐110	Association between SNP loci in FcɛR1β and CSU and to see its relation with serum IgE levels in Kashmiri population	No significant difference was found between the study population and control group in genotype distribution (wild and variant) among FcɛR1β loci (*p* value = 0.06, odds ratio = 0.29) Carriers of FcɛR1β (T allele) had a more significant risk of developing CAU than those with C allele (*p* = 0.01) Total IgE levels did not show any association with FceR1 gene polymorphisms	There is statistically no significant association between FcɛR1β gene polymorphism and CSU in Kashmiri population
Palikhe et al., 2015^43^	South Korea	Cross‐sectional	409 CU patients 388 healthy controls	Mean age‐39 years Males‐162 Females‐234	To investigate associations between PTPN22 gene polymorphisms and CU characteristics, including serum specific IgE antibodies response to toxic shock syndrome toxin‐1 (TSST‐1) and staphylococcal enterotoxin A (SEA)	Five PTPN22 single nucleotide polymorphisms, −1123G>C, 1858C>T, 13145A>G, 14943C>T, and 20628A>G, were genotyped. The genotype or haplotype frequencies of these polymorphisms were comparable between the 2 groups CU patients carrying the GG genotype at 20628A>G (*p* = 0.035) or haplotype 3 [GGG] (*p* = 0.047) had a significantly higher prevalence of serum specific IgE to TSST‐1 compared to non‐carriers CT/TT genotype at 14943C>T had a significantly higher prevalence of serum specific IgE to SEA (*p* = 0.045)	PTPN22 gene polymorphisms at 20628A>G and 14943C>T may enhance serum specific IgE responses to TSST‐1 and SEA, which may contribute to CU
Soltani et al., 2016^44^	Iran	Cross‐sectional	93 CIU patients and 93 healthy controls	NA	Investigate any relationship between genetic polymorphisms of FLG (filaggrin) and chronic idiopathic urticaria (CIU) Five single nucleotide polymorphisms (SNPs) of FLG were investigated: rs2485518, rs3126065, rs2786680, rs3814300, and rs3814299	All CIU patients and control subjects exhibited one given allele and consequently one given genotype as following: A/A genotype for two SNPs, rs3126065 and rs2786680, C/C genotype for two SNPs, rs2485518 and rs3814300, and G/G genotype for one SNP rs3814299 of FLG	None of five investigated SNPs (rs2485518, rs3126065, rs2786680, rs3814300, and rs3814299) of FLG gene are correlated with CIU in an Iranian population
Movahedi et al., 2017^45^	Iran	Cross‐sectional	90 patients with CSU 140 age‐sex matched healthy controls	NA	To evaluate the association of Th1 cytokines; IL‐2, IL‐12 and IFN‐γ polymorphisms with CSU	SNP (haplotype GT) at position −330 (and +166 of IL‐2 gene are differently expressed in CSU)	IL‐2 gene polymorphisms may be pathogenic for CSU, but there is no role of IL‐12 and IFN‐γ gene polymorphisms in the Iranian population
Brzoza et al., 2017^46^	Poland	Cross‐sectional	149 patients with ASST+ CSU 100 healthy controls (HCs)	NA	To investigate the role of polymorphisms in the genes for CD28 and inducible T‐cell costimulator (ICOS) in CSU pathogenesis In all subjects, the CD28 rs2140148 and rs3116496 and the ICOS rs6726035 polymorphisms were analysed	Statistically significant lower prevalence of the ICOS rs6726035 TT genotype among CSU patients (vs. HCs) The haplotypes rs2140148A, rs3116496T and rs6726035C presented a possible association with CSU No association of any polymorphism with disease characteristics	ICOS gene is more strongly associated with CSU pathogenesis, compared to the CD28 gene
Gimenez‐Arnau et al., 2017^47^	Spain	Cross‐sectional	20 patients with severely active CSU 10 healthy controls (HC)	Mean age‐51.5 15.7 years Males‐6 Female‐14	To identify relevant genes expressed in nonlesional (NLS‐CSU) and lesional skin (LS‐CSU) and peripheral blood of CSU patients, and compare with healthy controls	39 genes were differentially expressed in NLS‐CU, compared to HCs. Among them 31 (79.48%) genes were involved in epidermal homoeostasis and dermal repair 103 genes were differentially expressed in LS‐CSU, compared to NLS‐CSU. The genes involved in wheal formation contributed to several biological functions such as epidermal differentiation, intracellular signal function, transcriptional factors cell cycle differentiation, inflammation, or coagulation	The skin of CSU patients with a severely active disease shows an overall immunological genetic signature
Nasiri‐Kalmarzi et al., 2018^48^	Iran	Cross‐sectional	110 CU patients and 110 healthy controls	Mean age‐35 years Males‐40 Females‐70	Contribution of SNPs in the vitamin D receptor (VDR) and vitamin D binding protein (VDBP) genes in CU	Frequency of VDR rs1544410 AA genotype was higher in CU patients than controls, and it was associated with CU progression VDR rs2228570 and VDBP rs7041 SNPs had no statistically significant correlation with development or progression of CU	VDR rs1544410 polymorphism is a significant determinant of CU risk
Luo et al., 2018^49^	China	Cross‐sectional	24 children with autoimmune CSU and 24 control subjects	Mean age‐10 years Males‐11 Females‐13	Role of Oncostatin M receptor (OSMR) gene in autoimmune CSU via regulation of the JAK/STAT3 signalling pathway	Higher OSMR positive expression rate and elevated expression of the JAK/STAT3 signalling pathway‐related genes were observed in autoimmune CSU skin tissues	OSMR gene is highly expressed in autoimmune CSU. It causes upregulation of the JAK/STAT3 signalling pathway‐related genes, and may serve as a therapeutic target
Qu et al., 2019^50^	China	Cross‐sectional	53 CSU patients and 40 healthy volunteers	Mean age‐34 years Males‐25 Females‐28	Effect of miR‐194 on inflammatory reaction, mast cell degranulation, histamine release rate, endothelial cell permeability, and the expression of THBS1, IFN‐γ, TGF‐β, Smad3, and interleukin 4 in CSU patients	There was significantly higher expression of THBS1, TGF‐β, and Smad3 in the CSU group, while expression of miR‐194 and IFN‐γ were decreased In‐vitro transfection of cells with miR‐194 led to reduced mast cell degranulation, histamine release rate, and endothelial permeability, accompanied by an alleviated inflammatory reaction	miR‐194 negatively modulates THBS1 and inhibits the activation of TGF‐β/SMAD pathway, thereby alleviating the inflammatory response in CSU
Deza et al., 2019^51^	Spain	Single‐group cross‐sectional	50 patients with acquired cold urticaria (ACU)	Median age‐39 years Males‐16 Females‐34	To investigate the presence of variants in genes causing AIDs that present with cold‐induced urticarial skin rashes in patients clinically diagnosed with ACU, in order to look for susceptibility factors for the disease	Seven patients (14%) carried 8 heterozygous germline variants in the following genes: NLRP3 (*n* = 1), NLRP12 (*n* = 3), NLRC4 (*n* = 1), PLCG2 (*n* = 3) No pathogenic or likely pathogenic variants were detected, and no post‐zygotic variants were identified	ACU is not related to post‐zygotic or germline pathogenic variants in the NLRP3, NLRP12, NLRC4 and PLCG2 genes
Yan et al., 2019^52^	China	Single‐group, longitudinal trial	145 CSU patients	Age range‐18–66 years Males‐55 Females‐90	To investigate the impacts of CRP polymorphisms on the susceptibility and therapeutic efficacy of anti‐histamines in CSU patients	Patients with rs3093059TT genotype were significantly associated with good response to antihistamines, low CRP level Patients carrying the rs3093059C allele may respond poorly to mizolastine and have high CRP	CRP gene polymorphisms may predict the therapeutic response of CSU to antihistamines
Metz et al., 2019^53^	Germany	Randomised, double‐blind, placebo controlled trial	30 CSU patients (20 receiving omalizumab SC q4 wk, and 10 receiving placebo for 12 weeks), 10 untreated healthy volunteers (HVs)	NA	To study the effect of omalizumab on gene expression in skin biopsies from CSU patients in a double blind, placebo‐controlled design Lesional and nonlesional skin biopsies were collected for genetic analyses from the same area of consenting patients and assessed at baseline and on day 85 compared with skin biopsies from the same area of 10 untreated healthy volunteers (HVs)	At baseline, 63 transcripts were differentially expressed between lesional and nonlesional skin. Two‐thirds of these lesional signatures were also differentially expressed between lesional and HV skin Upon treatment with omalizumab, >75% of lesional signatures changed to reflect nonlesional skin expression levels (different vs. placebo, *p* < 0.01) Transcripts upregulated in lesional skin (vs. nonlesional and/or HV skin) suggested increased mast cell/leucocyte infiltration (FCER1G, C3AR1, CD93, S100A8, and S100A9), increased oxidative stress, vascularisation (CYR61), and skin repair events (KRT6A, KRT16) Lesional signatures were not modulated by treatment in nonresponders (defined based on UAS7 longitudinal changes ≥16)	Omalizumab, in treatment responders, reverted transcriptional signatures associated with CSU lesion phenotype to reflect nonlesional/HV expression levels
Dogan et al., 2020^54^	Turkey	Cross‐sectional	40 CSU patients and 40 healthy individuals	Mean age‐37 years Males‐12 Females‐28	Association between major histocompatibility complex class I and II alleles (HLA‐A, B, C, DRB and DQB), and CSU	HLA‐A*03 was significantly more frequent in the control group HLA‐DRB1 was more common in the CSU group, although statistically insignificant	HLA‐DRB1 may have a tendency to CSU, while HLA‐A plays a protective role
Nada et al., 2020^55^	Egypt	Cross‐sectional	70 CSU patients and 30 healthy controls	Mean age‐28 years Males‐29 Females‐41	Association of IL17RA gene with CSU susceptibility and severity	Carriers of rs4819554*G were at higher risk of CSU; rs4819554*A allele was associated with more severe phenotypes	IL17RA gene polymorphisms may increase CSU susceptibility and severity
Ma et al., 2020^56^	China	Cross‐sectional	90 CSU patients 90 healthy controls	Mean age‐35 years Males‐35 Females‐55	To explore the relationship between the vitamin D receptor (VDR) gene polymorphism and CSU Four VDR polymorphisms (TaqI, BsmI, ApaI, and FokI) were studied in all subjects	The mutant allele (C) of FokI was more common in CSU patients, compared to healthy controls (*p* = 0.02) Serum vitamin D levels were significantly lower in CSU patients than in healthy controls (*p* = 0.023)	VDR gene FokI (rs2228570) polymorphism plays a role in CSU pathogenesis in the Han Chinese population
Li et al., 2020^57^	China	Single‐group, longitudinal trial	114 CSU patients	Mean age‐56 years Males‐45 Females‐69	To evaluate the association between HRH1 gene rs901865 polymorphism and the severity of sedation side effect following desloratadine therapy (5 mg/day for 4 weeks) in CSU patients	17.50% (20/114) patients showed sedation side effect after desloratadine treatment The frequency of HRH1 rs901865 G allele was significantly higher than rs901865 A allele in patients who experienced sedation. (*p* = 0.0009) Patients with the rs901865 G/G genotype suffered a more serious sedation side effect than patients with the rs901865 G/A genotype (*p* = 0.005)	HRH1 rs901865 allele may be utilised as a biomarker for predicting the severity of sedation side‐effect in CSU patients receiving desloratadine
Brzoza et al., 2020^58^	Poland	Cross‐sectional	153 autoreactive CSU patients 104 healthy controls	Mean age‐36 years Males‐62 Females‐91	To search for an association of IL‐1 gene polymorphisms and the pathogenesis of CSU	Prevalence of IL‐1 rs1304037 TT genotype and T allele was significantly higher in CSU patients (vs. Controls). [*p* = 0.005] Prevalence of IL‐1 rs1800587 GG genotype and G allele was significantly higher in the patient group (*p* = 0.0004) No association noted between IL gene polymorphisms and disease severity or age of onset	IL‐1 gene polymorphisms may significantly impact the susceptibility to CSU
Sadr et al., 2021^59^	Iran	Cross‐sectional	93 CSU patients and 100 healthy individuals	NA	The frequency of alleles, genotypes, and haplotypes of 5 SNPs of PTPN22 gene (rs12760457, rs2476601, rs1310182, rs1217414, and rs33996649) in CSU was investigated	Prevalence of the rs1310182*T allele was significantly higher among patients The rs1310182 CC and TT genotypes were more common in patients Haplotype analysis showed significant association of CGCGC, CGTGC, and TGCGC with CSU	PTPN22 gene polymorphisms may be associated with an increased susceptibility to CSU
Khoshkhui et al., 2021^60^	Iran	Cross‐sectional	100 CSU patients, 100 age‐sex matched healthy controls	Mean age‐35 years Males‐28 Females‐72	Compare the frequency Taq1 polymorphism in the VDR gene between CSU patients and healthy controls	There was a significant relationship between Taq1 gene polymorphism (tt genotype) and CSU (*p* = 0.038) The risk of CSU was maximally increased by tt genotype (odds ratio = 4.6), followed by Tt genotype (odds ratio = 1.596) The mean serum vitamin D level in the CSU group was not significantly correlated with the Taq1 polymorphism (*p* = 0.8)	The frequency of Taq1 genotype polymorphism in the VDR gene was significantly higher in patients with CSU compared to the control group The tt and Tt genotypes polymorphism may be risk factors for CSU
Qi et al., 2021^61^	China	Case‐control	95 CSU patients and 95 age‐, sex‐ and ethnicity‐matched controls	Mean age‐36 Males‐26 Females‐69	Genome‐wide DNA methylation profile in whole blood of CSU patients	Average global DNA methylation levels of the 439 identified differentially methylated positions (DMPs) in CSU patients were significantly lower than controls Of the 28 differentially methylated genes (DMGs), HLA‐DPB2, HLA‐DRB1, PPP2R5C, and LTF were associated with autoimmunity	Possible role of epigenetics in CSU pathogenesis
Petruk et al., 2022^62^	Ukraine	Cross‐sectional	12 patients with acute urticaria (AU) 12 healthy controls	NA	To detect and verify innate and adaptive immune responses pathway‐focussed genes expression in patients suffering from AU and control group A pathway‐specific polymerase chain reaction PCR array was conducted using peripheral blood mononuclear cells (PBMCs) from patients and controls	Genes associated with AU development and pathogenesis→ *Increased transcriptional activity*—CD40, CD40LG, CD80 (B7‐1), C‐reactive protein, myeloperoxidase genes, Interferon gamma, interleukin (IL4), IL5, IL17A, tumour necrosis factor, chemokine CXCL8, RAR‐related orphan receptor C Th17 differentiation regulator, the NLRP3 inflammasome genes, and the NFKB1 transcription factor *Reduced transcriptional activity*—FOXP3 gene and the suppressor cytokine IL10 linked to T‐reg	AU is associated with transcriptional activation of pro‐inflammatory genes, and concomitant suppression of anti‐inflammatory genes Both innate and adaptive immune pathways contribute to AU pathogenesis
Egea et al., 2022^63^	Columbia	Cross‐sectional	100 patients with CSU 100 healthy controls	NA	To explore the role of VDR SNPs (*TaqI, BsmI, FokI, and ApaI genes*), and serum vitamin D levels in Colombian Caribbean CSU patients	G allele in TaqI (OR 2.1) and A allele in FokI SNPs (OR 1,6) of VDR gene were significant risk‐factors for CSU (vs. healthy controls) (*p* < 0.05) GCCA haplotype showed decrease in vitamin D levels (11.34 ng/mL; *p* = 0.002) with the G allele of TaqI and A allele of FokI gene SNPs	TaqI [rs731236] and FokI [rs2228570] VDR gene SNPs increased the risk for CSU
Prosty et al., 2022^64^	Canada	Cross‐sectional	15 CSU patients (lesional and non‐lesional), 12 healthy control (HC) skin biopsies, 20 CSU and 10 HC blood samples	NA	To investigate the role of Th2 and Th17 pathway genes in CSU pathogenesis by re‐analysing publicly available transcriptomic data	Th2 (IL‐4/13 signalling) and Th17‐related (IL‐17/23 signalling) genes were upregulated in lesional compared to non‐lesional and HC samples Th2 scores CSU patients correlated positively with disease severity Increased regulatory T‐cells (Treg) and resting mast cells in non‐lesional skin Th1 scores were not significantly different between lesional and HC samples	Th2 and Th17‐related genes and pathways are involved in CSU pathogenesis Th2 scores associate with disease severity, which underlie their clinical relevance
Andrades et al., 2022^65^	Spain	Cross‐sectional	45 patients with moderate‐to‐severe CSU 17 healthy controls (HCs)	NA	To study the expression of PAFR (platelet activating factor receptor) gene in active wheal (lesional skin‐CSU), non‐lesional CSU skin and healthy controls	PAFR mRNA expression was significantly higher in LS‐CSU versus HCs (*p* = 0.014) PAFR positive staining in immunohistochemistry was mainly found in the epidermal basal layer in HCs, whereas it was broadly present along the epidermis in LS‐CSU samples Endothelial cells showed PAFR expression exclusively in LS‐CSU and NLS‐CSU samples	PAFR is differentially expressed in CSU patients versus HCs, suggesting an active role of PAF in wheal formation Additionally, significantly lower serum PAF‐AH (acetylhydrolase)/PAF ratio in sgAH non‐responders versus responders suggests that PAF could be a potential biomarker of sgAH refractoriness
Fang et al., 2023^66^	China	Record‐based study from gene expression omnibus (GEO) database of NCBI	Two microarray datasets from the GEO database of NCBI were merged and validated‐GSE57178 and GSE72540 They included skin samples from three groups: Lesional (CSU‐L) and non‐lesional skin (CSU‐C) of patients with active CSU, as well as from healthy volunteers (Nor)	NA	To identify key genetic factors and investigate their roles in CSU pathogenesis	Chemokine (C‐C motif) ligand 2 (CCL2) and cholesterol 25‐hydroxylase (CH25H) gene, and tumour necrosis factor (TNF) signalling pathways play crucial role in CSU pathogenesis Knockdown of CCL2, CH25H, and TNF genes reduced excitability and cytokine production in human mast cells	Genes involved in the CCL2, CH25H, and TNF pathways play crucial roles in CSU pathogenesis and may become potential therapeutic targets
Liu et al., 2023^67^	China	Record‐based study from gene expression omnibus (GEO) database of NCBI	Microarray datasets of skin tissue from CSU patients and healthy controls obtained from Gene Expression Omnibus database	NA	Identification of hub genes and correlation of mRNA expression of 14 identified hub genes with clinical parameters in CSU	Positive correlation of mRNA expressions of S100A7, S100A8, S100A9, S100A12, IL6 and SOCS3 were found with 7‐day urticaria activity score (UAS7) in CSU The hub genes PVT1, SNHG3 and ZBTB20 − AS1 have potential diagnostic value for CSU IL‐6/miR − 149 − 5p/ZBTB20 − AS1 axis may have an important role in the activation of mast cells in CSU	IL‐6 and its related regulatory molecules may be used as potential diagnostic markers and therapeutic targets for CSU
Chang et al., 2023^68^	United States	Genome‐wide association study (GWAS)	679 CSU patients and 4446 controls	NA	Genome‐wide association study to investigate the genetic susceptibility of CSU	2 loci were identified to be significantly associated with risk of CSU These loci map to the MHC region (strongest association for HLA DQ A1) and a region near ITPKB Polygenic scores for 3 autoimmune‐related disorders (hypothyroidism, type 1 diabetes, and vitiligo) were associated with CSU risk and CU index (*p* < 0.002, odds ratio >1.72)	Specific genetic components (HLA and ITPKB) may be associated with the risk of CSU and autoimmunity
Su et al., 2023^69^	China	Record‐based study from gene expression omnibus (GEO) database of NCBI	Two microarray data sets with mRNA information of skin from CSU patients and healthy controls obtained from Gene Expression Omnibus database	NA	Identification of hub genes and key genetic drivers of CSU pathogenesis	92 up‐regulated and 7 down‐regulated genes were identified in CSU lesions Three signalling pathways (TNF, NF‐kB, and JAK‐STAT), and four hub genes (IL‐6, TLR‐4, ICAM‐1, and PTGS‐2) were significantly linked to CSU pathogenesis and the latter correlated with immune and stromal cell infiltration in lesional skin	TNF, NF‐kB, JAK‐STAT, IL‐6, TLR‐4, ICAM‐1, and PTGS‐2 may be potential targets for novel CSU treatments
Zhang et al., 2023^10^	China	Genome‐wide association study (GWAS)	800 CSU cases and 900 healthy controls	Mean age‐37.5 years Males‐355 Females‐875	Identification of susceptibility SNPs associated with CSU and evaluation of their association with autoimmunity or atopy	Five SNPs (rs434124, rs61986182, rs73075571, rs9378141 and rs3789612) were identified to be associated with CSU Significant associations were observed between allele frequencies of the identified SNPs and autoimmune‐related CSU phenotypes	CSU, especially autoimmune CSU, has a hereditary component and shares genetic overlap with other autoimmune diseases, but not atopic diseases
Hussain et al., 2023^70^	India	Cross‐sectional	154 atopic individuals (including 52 CU patients) and 160 healthy controls	NA	Effect of SOCS5 SNP (rs41379147) on allergic disorders, and its relationship with various clinicopathological parameters in atopic diseases	There is no statistically significant difference in the SOCS5 genotype distribution and allele frequency between the patients and the healthy controls, with respect to either overall atopic diseases or in individual subgroups, including CU	SOCS5 SNP rs41379147(C/T) does not significantly increase the risk of developing any allergic disorder
Chen et al., 2024^71^	China	Record‐based study from gene expression omnibus (GEO) database of NCBI	Differentially expressed genes between skin lesions of CSU and normal controls (LNS‐DEGs) were identified, and the enrichment analyses of LNS‐DEGs were performed	NA	To explore the underlying genetic mechanisms of CSU and identify potential therapeutic targets	Among 247 LNS‐DEGs, seven genes were upregulated (PTGS2, CCL2, IL1B, CXCL1, IL6, VCAM1, ICAM1) and one downregulated hub gene (PECAM1). (CSU vs. healthy controls) PTGS2, encoding cyclooxygenase 2 (COX2), demonstrated strongest association with CSU COX2 inhibitor, celecoxib, significantly inhibited mast cell degranulation, and reduced vascular permeability and inflammatory cytokine expression in mouse model	PTGS2 gene may be a potential regulator of immunity and inflammation in CSU Targeting PTGS2 by COX‐2 inhibitors is a potential therapeutic option in CSU
Guo et al., 2024^72^	China	Record‐based study from gene expression omnibus (GEO) database of NCBI	Microarray datasets of samples from CSU patients and healthy controls obtained from Gene Expression Omnibus database	NA	Identification of key genes and molecular mechanisms of CU	27 hub genes were identified to be implicated in CU pathogenesis, including interleukin‐6 (IL‐6), prostaglandin‐endoperoxide synthase 2 (PTGS2), and intercellular adhesion molecule‐1 (ICAM1)	The identified hub genes are critical in the pathogenesis of CU coupled with signalling pathways of TNF, JAK‐STAT, and NF‐kB
Li et al., 2024^73^	China	Cross‐sectional	159 patients with acute urticaria and 1745 healthy individuals—30 in each group were subjected to genetic testing	Overall: Median age‐ 28 Males‐68 Females‐91 AU patients who underwent genetic testing: Mean age‐30 Males‐12 Females‐18	Assessment of expression of genes encoding dsDNA receptors, ssRNA receptors, exosome formation, and type I interferon in the peripheral blood of patients and controls	AU patients had significantly higher serum dsDNA levels and upregulated expression of genes encoding dsDNA receptors (LRRFIP1, POLR3A, MRE11, and TLR3), ssRNA receptors (TLR8), absent in melanoma factor 2 (AIM2)‐related inflammatory factors (CASP1, IL‐1β), and interferon α and β	dsDNA may participate in the pathogenesis of acute urticaria
Smola et al., 2025^74^	Germany	Cross‐sectional	17 patients with antihistamine refractory CSU 8 healthy controls (HCs)	Age range‐19–69 years Males‐6 Females‐11	To identify whether gene expression was altered by omalizumab therapy (responders vs. non‐nonresponders)	Several genes were overexpressed in CSU patients (vs. HCs) such as CD52, CD28, COMMD6, and CLEC2B‐ most involved with T‐cell pathway Several miRNAs were overexpressed in CSU patients (vs. HCs)‐ hsa let‐7e‐5p, hsa‐miR‐3609, and hsa‐miR‐486‐3p The comparison between responders, nonresponders, and healthy controls showed significant differences for hsa‐miR‐3609 between healthy controls and responders and for hsa‐miR‐486‐3p between healthy controls and both responders and nonresponders	Omalizumab therapy altered the mRNA and niRNA profile of CSU patients The greatest changes in expression levels were observed on day 2 following the first omalizumab dose

The included studies ranged from familial case reports to GWAS. Recent research, has utilised genomic data to identify candidate genes involved in urticaria pathogenesis. Multiple genes have been implicated so far in increasing the susceptibility and severity of various types of urticaria. Mutations in some of these genes may serve as biomarkers for specific disease phenotypes, predict treatment responses, or represent potential therapeutic targets for precision medicine (Table 2).

**TABLE 2:** Genetic variants and polymorphisms in CU.

Genes associated with increased risk of CU	Genes with a protective role in CU	Genes without significant association with CU
HLA genes		
B44^25^, ^29^, ^31^ Bw4^26^ DRB1*12^24^ DRB1*0901^24^ DRB1*04^22^ DRB1*01^29^ DRB1*15^29^ DQ1^26^ DQA1^68^ DQB1*0302^22^ DR4^23^	A*03^54^ A24^26^ A33^31^ Cw4^25^ Cw7^25^ DQB1*05^24^ DRB1*15^52^ DQB1*06^52^	A, B, DRB1^36^
Genes encoding inflammatory mediators and related proteins		
IgE (Cε)^20^ FCER1A SNP rs2298805^41^ C4B*Q0^19^ TGF‐β1 gene promoter (−509C>T)^27^ TGF‐β (codon 10G/A and 25G/C)^38^ TNF (G/A‐308, G/A‐238)^9^ IL‐1 polymorphisms rs1304037, rs1800587^58^ IL‐2 SNP (GT at −330 and + 166)^45^ IL‐6 (G/C‐174, G/A nt565)^9^ IL‐10 (AG‐1082, CT‐819, and CA‐592)^38^ IL17RA rs4819554*G^55^ CD28 rs2140148A/rs3116496T, ICOS rs6726035C^46^ CCR2 A^40^ COX‐1 22 C/T, COX‐2 5_UTR T/G, COX‐2 Exon 10 T/C^32^ FLAP‐336 G/A^32^	CCR5 d32^40^	FCER1B^42^ IL‐12 SNP (A/C −1188)^45^ IFNG (A/T UTR5644)^33^ SOCS5 SNP rs41379147^70^ CTLA‐4 A49G^39^
Others		
PTPN22 SNP rs2488457C, rs1310182T, rs3811021T^33^, ^59^ VDR FokI, Taq1 polymorphisms^48^, ^56^, ^60^, ^63^ ITPKB^68^ Platelet activating factor (PAF)^65^		PDCD‐1 (PD1.3 and PD1.5 polymorphisms)^35^ FLG SNPs rs2485518, rs3126065, rs2786680, rs3814300, rs3814299^44^ ACE I/D polymorphisms^28^

The findings have been organised into three subgroups for a better understanding of this complex topic.

#### 3.1.1 Role in pathogenesis

##### Familial predisposition and heritability

The genetic basis of urticaria parallels other allergic diseases such as atopic dermatitis, asthma, and allergic rhinitis, which show familial clustering. Studies indicate a lifetime risk 8.8 times higher for individuals with both affected parents compared to those without a family history.^75^ A study done on 1480 Swedish twin children reported heritability of 54% in boys and 60% in girls, with environmental factors also contributing.^21^

Kai and Flohr^37^ described aquagenic urticaria in monozygotic twins, thus suggesting a genetic background. Case reports have documented autosomal dominant inheritance patterns in familial forms such as chronic familial giant urticaria and dermo‐distortive urticaria.^16^, ^18^

##### Genetic mutations and polymorphisms association

Multiple studies have evaluated the association of urticaria with various genes, especially those involved in immunological pathways, thus influencing the pathophysiology of the disease. Majority of these studies involved patients with CU, especially CSU. Table 2 shows the various genes that have been implicated in increasing the risk of CU, and those that may play a protective role.

###### HLA genes

Among HLA genes, HLA‐ B, HLA‐DRB, HLA‐DQA, HLA‐DQA1 and HLADR4 have been significantly associated with CU by several authors.^22^, ^23^, ^24^, ^25^, ^26^, ^29^, ^31^, ^68^ Furthermore, Chang et al.^68^ also highlighted that polygenic scores for 3 autoimmune‐related disorders (hypothyroidism, type 1 diabetes, and vitiligo) were strongly associated with CSU risk and CU index (*p* < 0.002, OR >1.72). On the other hand, HLA‐A, HLA‐Cw and HLA‐DQB may confer reduced susceptibility to CU.^22^, ^24^, ^25^, ^26^, ^31^, ^54^ A single study found no HLA‐A, B or DRB1 association with ASST‐positive CSU.^36^

These loci map to the major histocompatibility complex (MHC) region (strongest association for HLA DQ A1) and a region near ITPKB.

Polygenic scores for three autoimmune‐related disorders (hypothyroidism, type 1 diabetes, and vitiligo) were associated with CSU risk and CU index (*p* < 0.002, OR >1.72).

###### IgE pathway genes

Polymorphisms affecting IgE levels and receptor interactions have been implicated in CSU susceptibility. The high‐affinity IgE receptor is expressed on the surface of the mast cells and basophils, the primary effector cells in urticaria. Polymorphisms of the genes encoding for IgE and the α‐subunit of the IgE receptor have been found to affect serum IgE levels and subsequently increase the risk of acquiring CSU.^20^, ^41^ However, no significant association between FcɛR1β gene polymorphism and CSU was observed in an Indian study.^42^

###### Complement, arachidonic acid metabolites and cytokine genes

The role of genes encoding various inflammatory mediators has been evaluated by a few authors. In a study by Gell et al.,^19^ loss‐of‐function mutation in the C4 gene leading to C4 deficiency was significantly associated with multiple disorders including urticaria and angioedema. Another study implicated variations in genes encoding arachidonic acid metabolites in the pathogenesis of CSU.^32^ Mast cell activation, in addition to release of histamine and leukotrienes, produces pro‐inflammatory cytokines, which result in progression of inflammation in CU. On the other hand, regulatory mechanisms help mitigate the inflammatory process through anti‐inflammatory cytokines. Variations in the respective genes encoding proteins involved in the inflammatory pathways cause alteration in the cytokine profile. Thus, mutations in genes encoding proteins involved in both pro‐inflammatory pathways like IL‐1, IL‐2, IL‐6, TNFα and IL17 receptor, as well as anti‐inflammatory pathways like TGF‐β and IL‐10 have been shown to be associated with CSU.^9^, ^38^, ^45^, ^55^, ^58^ Furthermore, IL17RA rs4819554*A polymorphism was associated with more severe disease in terms of more prolonged disease activity, concurrent angioedema, requirement of higher level of treatment, and worse quality of life scores.^55^ Interestingly, Movahedi et al. observed no significant association of SNPs of IL‐2 and IFNG with CSU.^45^

Brzoza et al. investigated the contribution of genetic polymorphisms of chemokine receptors and co‐stimulatory molecules involved in T‐cell mediated inflammatory response in autoimmune CSU. While CCR‐2, ICOS and CD28 polymorphisms increased susceptibility to autoimmune CSU, CCR‐5 32 base pair deletion was shown to significantly reduce the risk.^40^, ^46^ Another such molecule, CTLA‐4, which has been implicated in the pathogenesis of autoimmune thyroiditis, appeared to play no role in CSU.^55^

###### Other genes

Among other genes, Protein tyrosine phosphatase‐22 (PTPN22) has been studied by several authors in relation to urticaria. This gene is considered to have a strong association with autoimmunity. SNP 1858C>T of PTPN22 has been linked to several multisystem autoimmune disorders including systemic lupus erythematosus, rheumatoid arthritis, type 1 diabetes, Graves disease and Hashimoto thyroiditis. Although the same SNP appeared to have no role in CU pathogenesis, three distinct polymorphisms of the gene (rs2488457C, rs1310182T and rs3811021T) have been found to be significantly associated with CSU, particularly the autoimmune subtype.^33^ Palikhe et al.^43^ investigated five SNPs of PTPN22 and found comparable results between CSU patients and controls. However, they found two SNPs 20628A>G and 14943C>T to enhance specific IgE responses to staphylococcal superantigens, which may in turn contribute to the development of CU.

Brzoza et al.^35^ evaluated polymorphisms in another gene, programed cell death 1 gene, in autoimmune CU patients. This gene encodes a T‐cell surface receptor, which plays an important role in the suppression of autoreactive responses by inhibition of cytokine production and subsequent T‐cell over‐proliferation. Polymorphisms in the gene result in reduced expression of the molecule, leading to lymphocyte hyperactivity. Several diseases have been linked to PD1.3 and PD1.5 polymorphisms, including type 1 diabetes mellitus, lupus nephritis, rheumatoid arthritis, and ankylosing spondylitis. However, no significant association was established with autoimmune CU.

Variations in several other genes, such as SOCS5, FLG and angiotensin‐converting enzyme, are known to increase the susceptibility of other multisystem immune‐related disorders, but their role in urticaria remains to be identified.^70^ Insertion/deletion polymorphism in the latter, though not associated with increased risk of CSU, may be involved in the association of angioedema with CSU.^28^

In the case of CIndU, there is a single study on 50 patients with acquired cold urticaria. Unlike familial cold autoinflammatory syndrome (previously known as familial atypical cold urticaria), these patients were not associated with any post‐zygotic or germline pathogenic variants in the NLRP3, NLRP12, NLRC4 and PLCG2 genes.^51^

Recently, Andrades et al.^65^ speculated the role of platelet activating factor in CU pathogenesis by detecting higher expression of PAF receptor gene in CU patients, compared to healthy controls (HCs).

Studies on genetic variations and polymorphisms associated specifically with AU have not been conducted. Brachtel et al. compared the phenotype and gene distribution of 15 blood group polymorphisms between 239 atopic patients (including 61 patients with AU) and 151 HCs. They found significant distribution differences in the polymorphic systems Kidd, Inv, and red cell acid phosphatase.^17^

###### Vitamin D receptor (VDR) gene

Vitamin D plays an immunomodulatory role in various diseases and is known to regulate innate and adaptive immune pathways. Its activity is mediated by attaching to vitamin D receptor (VDR), which further leads to activation of the retinoid X receptor. It can induce apoptosis, and regulate cell differentiation, macrophage activity, and survival of mast cells. Moreover, it leads to inhibition of both T‐cell and B‐cell responses. SNPs of the *VDR* gene have been implicated in various diseases, including rheumatoid arthritis, diabetes, and malignancy.^63^ In CU patients, significant association of SNP rs1544410, FokI (rs2228570) and Taq1 (rs731236) polymorphisms were observed across multiple studies.^48^, ^56^, ^60^, ^63^

###### Alteration in gene expression and epigenetics

The gene expression pattern in the skin of patients with urticaria differs from that of healthy skin. Urticarial skin shows upregulation of genes related to inflammatory mediators and pro‐inflammatory cytokine signalling pathways, whereas regulatory genes usually have reduced expression. A Spanish study corroborates this fact which detected higher expression of immunological pathway genes in the lesional skin (wheals and hives) of severely active antihistamine refractory CSU patients by transcriptome analysis compared to non‐lesional skin of CSU patients and HCs.^47^ In addition, epigenetic modifications altering genetic expression have also been identified in these patients. In recent years, several GWAS and record‐based studies from genomic databases have been carried out to identify hub genes that are more commonly associated or upregulated in patients with urticaria. Guo et al.^72^ identified 27 hub genes implicated in CU pathogenesis, including interleukin‐6 (IL‐6), Prostaglandin‐endoperoxide synthase 2 (PTGS2), and intercellular adhesion molecule‐1 (ICAM1). Majority of these studies have been conducted on CSU patients, with only two studies on patients with AU. The summary of epigenetic and gene expression studies is tabulated in Table 3.

**TABLE 3:** Summary of studies on gene expression and epigenetics in urticaria.

Authors	Study category	Urticaria subtype	Findings and remarks
Luo et al., 2018^49^	Gene expression study based on immunohistochemical analysis on skin samples	Autoimmune CSU	Affected skin showed higher expression of Oncostatin M receptor (OSMR) and genes involved in JAK/STAT3 signalling pathway
Qu et al., 2019^50^	Gene expression study based on data from Gene Expression Omnibus (GEO)	CSU	Upregulation of Thrombospondin‐1 (THBS1), TGF‐β, and Smad3 genes in CSU Downregulation of miR‐194, which modulates THBS1 and inhibits the activation of TGF‐β/SMAD pathway, thereby alleviating the inflammatory response In‐vitro transfection of cells with miR‐194 led to reduced mast cell degranulation, histamine release rate, and vascular permeability
Qi et al., 2021^61^	Epigenetic study	CSU	Genome‐wide DNA methylation profile showed significantly lower average methylation levels of 439 identified differentially methylated positions (DMPs) in CSU patients
Petruk et al., 2022^62^	Gene expression study based on PCR array using peripheral blood mononuclear cells (PBMCs)	AU	Increased transcriptional activity of pro‐inflammatory genes CD40, CD40LG, CD80 (B7‐1), C‐reactive protein, myeloperoxidase, interferon gamma, IL4, IL5, IL17A, TNF, CXCL8, RAR‐related orphan receptor C Th17 differentiation regulator, NLRP3 inflammasome genes, and NFKB1 transcription factor Reduced transcriptional activity of FOXP3 gene and the suppressor cytokine IL10
Prosty et al., 2022^64^	Gene expression study based on data from GEO	CSU	Th2 (IL‐4/13 signalling) and Th17‐related (IL‐17/23 signalling) genes were upregulated in lesional samples Increased regulatory T‐cells (Treg) and resting mast cells in non‐lesional samples
Fang et al., 2023^66^	Gene expression study based on data from GEO	CSU	Expression of Chemokine (C‐C motif) ligand 2 (CCL2), cholesterol 25‐hydroxylase (CH25H) gene, and TNF signalling genes correlated highly with CSU Knockdown of CCL2, CH25H, and TNF genes reduced excitability and cytokine production in human mast cells
Liu et al., 2023^67^	Gene expression study based on data from GEO	CSU	Positive correlation of mRNA expressions of S100A7, S100A8, S100A9, S100A12, IL6 and SOCS3 were found with UAS7 scores IL‐6/miR‐149‐5p/ZBTB20‐AS1 axis may have an important role in the activation of mast cells in CSU
Su et al., 2023^69^	Gene expression study based on data from GEO	CSU	92 up‐regulated and 7 down‐regulated genes were identified in CSU lesions Three signalling pathways (TNF, NF‐kB, and JAK‐STAT), and four hub genes (IL‐6, TLR‐4, ICAM‐1, and PTGS‐2) were significantly linked to CSU pathogenesis and the latter correlated with immune and stromal cell infiltration in lesional skin
Chen et al., 2024^71^	Gene expression study based on data from GEO	CSU	CSU was associated with upregulation of seven genes (PTGS2, CCL2, IL1B, CXCL1, IL6, VCAM1, ICAM1) and downregulation of 1 gene (PECAM1) PTGS2, encoding cyclooxygenase 2 (COX2), demonstrated strongest association with CSU
Guo et al., 2024^72^	Gene expression study based on data from GEO	CSU	27 hub genes were implicated in CU pathogenesis, including interleukin‐6 (IL‐6), prostaglandin‐endoperoxide synthase 2 (PTGS2), and intercellular adhesion molecule‐1 (ICAM1) Signalling pathways of TNF, JAK‐STAT, and NF‐kB play a critical role in CSU
Li et al., 2024^73^	Gene expression study based on quantitative PCR on peripheral blood samples	AU	AU patients had significantly higher serum dsDNA levels and upregulated expression of genes encoding dsDNA receptors (LRRFIP1, POLR3A, MRE11, and TLR3), ssRNA receptors (TLR8), absent in melanoma factor 2 (AIM2)‐related inflammatory factors (CASP1, IL‐1β), and interferon α and β

#### 3.1.2 Role in diagnosis

Our systematic review identified a single study which reported the potential diagnostic value of certain genes in CSU. Liu et al.^67^ identified a total of 296 differentially expressed genes in CSU patients compared to HCs. Among these DEGs, certain hub genes were identified viz. S100A7, S100A8, S100A9, S100A12, IL6 and SOCS3, whose mRNA expression correlated positively with the urticaria activity score and may have a potential diagnostic value. Additionally, Receiver Operating Characteristic analysis showed that the expression of PVT1, SNHG3 and ZBTB20 − AS1 genes may increase the predisposition for CSU, indicating a diagnostic role. These genes are components of the IL‐6 pathway, and therefore, IL‐6 expression may be considered a diagnostic biomarker for CSU.

#### 3.1.3 Role in therapeutics

Few authors have highlighted the potential role of genetics in therapy of chronic urticaria with antihistamines.

##### H1 antihistamines

Palikhe et al.^30^ reported that CU patients with polymorphism of the CRTH2 gene (−466T>C and −129C>A) showed significant differences in treatment response with antihistamines, but there was no association with disease characteristics, viz. duration, atopy and serum total IgE. CU patients with homozygous TT genotypes had significantly higher dose requirements of antihistamines than those with the CT and CC genotypes. In the same line Guo et al.,^41^ Zhou et al.^34^ and Yan et al.^52^ demonstrated that polymorphisms of the FCER1A and C‐reactive protein CRP genes may predict H1‐antihistamine effectiveness in CSU patients. Patients carrying the rs3093059TT allele of the CRP gene may respond favourably to non‐sedating H1 antihistamines such as desloratadine, mizolastine and fexofenadine, whereas the rs3093059C allele showed poor response to mizolastine.

##### Sedative effects of antihistamines

Li et al.^57^ conducted a very interesting study to identify genetic predictors for the extent of sedation following desloratadine therapy. They reported that the HRH1 gene polymorphism rs901865 is associated with increased sedation after desloratadine treatment in Chinese patients with CSU, and thus proposed its potential utilisation as a biomarker for predicting the severity of sedative side‐effects of desloratadine.

##### Omalizumab

Two recent studies have highlighted a link between genetic expression in CSU patients and omalizumab therapy. Metz et al.^53^ reported that omalizumab therapy reverted the expression of certain genes such as FCER1G, C3AR1, CD93, S100A8, and S100A9 in lesional skin of CSU patients to reflect the genetic signature of non‐lesional skin or HCs. This alteration was restricted to treatment responders, consistent with their clinical improvement. Additionally, omalizumab therapy may alter the expression of certain T‐cell and thrombocyte associated genes and miRNAs in CSU patients, although most of them remain unrelated to CSU pathogenesis.^74^

##### Gene‐targeted therapies

Recently, several authors have explored the gene expression omnibus (GEO) database of the NCBI to identify genes associated with the pathogenesis of CSU, which may be utilised as potential therapeutic targets. Fang et al.^66^ proposed that genes involved in the CCL2 (chemokine C‐C motif ligand 2), CH25H (cholesterol 25‐hydroxylase), and tumour necrosis factor (TNF) signalling pathways are intricately associated with CSU pathogenesis and may become potential therapeutic targets. Several other pathways and their regulatory genes have also been proposed as potential therapeutic targets, such as IL‐6, TNF, NF‐kB, JAK‐STAT, IL‐6, TLR‐4, ICAM‐1, and PTGS‐2.^67^ Chen et al.^71^ further proposed that celecoxib, the COX‐2 inhibitor, which targets the PTGS2 pathway, may be explored as a novel treatment option for refractory CSU as it significantly inhibited mast cell degranulation, and reduced vascular permeability and inflammatory cytokine expression in mouse model.

### 3.2 Meta‐analysis

#### 3.2.1 Genetic associations with chronic urticaria

The meta‐analysis included studies investigating the role of specific genetic factors and polymorphisms in chronic urticaria, conducted only when at least three studies provided sufficient and comparable data on a specific genetic factor related to urticaria, with a high level of consistency across studies required for pooling data.

##### HLA‐B44

**FIGURE 2:**
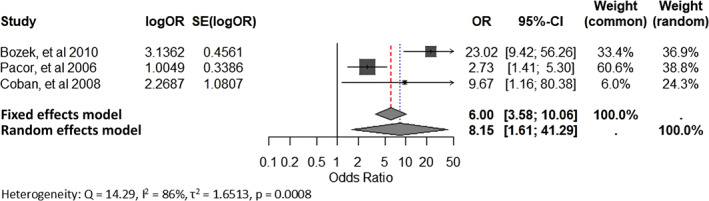
Forest plot summarizing the association between HLA‐B44 and chronic urticaria. For each study, the grey square represents the odds ratio (OR) and its horizontal line the 95% confidence interval (CI), with square area proportional to the inverse‐variance weight. The smaller grey diamond depicts the fixed‐effect summary estimate (OR = 6.00, 95% CI 3.58–10.06), whereas the larger grey diamond depicts the random‐effects summary estimate (OR = 8.15, 95% CI 1.61–41.29); the lateral tips of each diamond correspond to the pooled CI. The solid vertical line at OR = 1.0 denotes “no association,” the red dashed vertical line marks the fixed‐effect pooled OR, and the blue dotted vertical line marks the random‐effects pooled OR. Substantial between‐study heterogeneity was present (Q = 14.29, *I*² = 86%, *τ*² = 1.6513, *p* = 0.0008); therefore, the random‐effects summary estimate is considered the most appropriate overall effect size. The plot demonstrates a statistically significant positive association between HLA‐B44 carriage and increased susceptibility to chronic urticaria.

Three case‐control studies assessed the contribution of the HLA‐B44 allele to chronic urticaria susceptibility.^25^, ^29^, ^31^ The results identified HLA‐B44 as a shared genetic factor contributing to disease susceptibility, with a pooled OR of 8.15 (95% CI: 1.61–41.29), but with substantial between‐study heterogeneity (Q = 14.29, *I*² = 86%, *τ*² = 1.6513, *p* = 0.0008). Individual ORs were 9.67 (95% CI: 1.16–80.38) for Coban et al.,^29^ 2.73 (95% CI: 1.41–5.30) for Pacor et al.,^25^ and 23.02 (95% CI: 9.42–56.26) for Bozek et al.,^31^ highlighting considerable variation in effect size across populations (Figure 2). Therefore, the random‐effects estimate (OR = 8.15, 95% CI 1.61–41.29) is the preferred summary measure. Despite the wide confidence interval, this eight‐fold increase in risk confirms that HLA‐B44 is a statistically significant genetic marker for chronic urticaria, while the high heterogeneity suggests that the magnitude of association may differ by ethnicity, study design or environmental context.

##### VDR gene polymorphisms

###### FokI polymorphism

**FIGURE 3:**
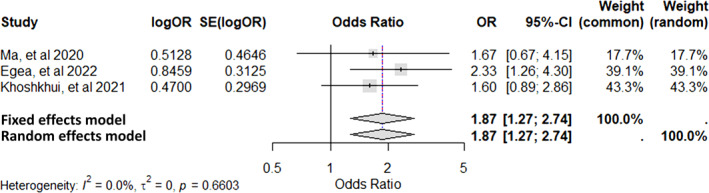
Forest plot summarizing the association between the FokI vitamin‐D‐receptor polymorphism (heterozygous genotype) and chronic spontaneous urticaria (CSU). For each study, the grey square marks the odds ratio (OR) and its horizontal line the 95% confidence interval (CI), with square size proportional to the study’s inverse‐variance weight. The grey diamonds represents the fixed‐effect summary estimate (OR = 1.87, 95% CI 1.27–2.74), and the random‐effects summary estimate (also OR = 1.87, 95% CI 1.27–2.74); the diamond’s lateral tips denote the pooled CI. The solid vertical line at OR = 1.0 indicates “no association,” the red dashed line marks the fixed‐effect pooled OR, and the blue dotted line marks the random‐effects pooled OR. Between‐study heterogeneity was negligible (Q = 0.83, *I*² = 0%, *τ*² = 0, *p* = 0.6603); consequently, the fixed‐ and random‐effects estimates coincide, and either can be interpreted as the overall effect size. The plot demonstrates a statistically significant positive association between carrying the heterozygous FokI genotype and increased susceptibility to CSU.

The analysis of the FokI polymorphism (rs2228570) harmonised the alleles across studies (T and C in Ma et al.^56^ and Khoshkhui et al.^60^ and A and G in Egea et al.^63^) as complementary strands, enabling consistent evaluation of the heterozygous genotype (TC/AG). The pooled OR was 1.87 (95% CI: 1.27–2.74) under the random‐effects model, with negligible heterogeneity (*I* ^2^ = 0.0%, *τ* ^2^ = 0.0, *p* = 0.6603) (Figure 3). The Trim‐and‐Fill funnel plot revealed no imputed studies, suggesting no evidence of publication bias. These findings indicate a robust and significant association between the FokI heterozygous genotype and CSU susceptibility.

###### TaqI polymorphism

**FIGURE 4:**
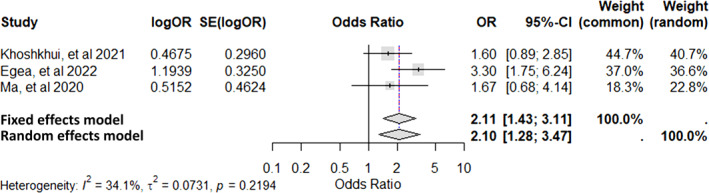
Forest plot summarizing the association between the TaqI vitamin‐D‐receptor polymorphism (heterozygous genotype) and chronic spontaneous urticaria (CSU). For each study, the grey square marks the odds ratio (OR) and its horizontal line the 95% confidence interval (CI); square size is proportional to the study’s inverse‐variance weight. The smaller grey diamond represents the fixed‐effect pooled estimate (OR = 2.11, 95% CI 1.43–3.11), while the larger grey diamond represents the random‐effects pooled estimate (OR = 2.10, 95% CI 1.28–3.47); the diamond’s lateral tips correspond to the pooled CI. The solid vertical line at OR = 1.0 denotes “no association,” the red dashed vertical line marks the fixed‐effect pooled OR, and the blue dotted vertical line marks the random‐effects pooled OR. Moderate between‐study heterogeneity was observed (Q = 3.04, *I*² = 34.1%, *τ*² = 0.066, *p* = 0.22); consequently, the random‐effects summary is considered the most appropriate overall estimate. The plot demonstrates a statistically significant positive association between carrying the heterozygous TaqI genotype and increased susceptibility to CSU.

For the TaqI polymorphism (rs731236), alleles (T and C in Khoshkhui et al.^60^ and Ma et al.,^56^ and A and G in Egea et al.^63^ were similarly harmonised). The heterozygous genotype (Tt/AG) yielded a pooled OR of 2.10 (95% CI: 1.28–3.47) under the random‐effects model. Moderate heterogeneity was observed (*I* ^2^ = 34.1%, *τ* ^2^ = 34.1, *p* = 0.2194) (Figure 4). Trim‐and‐Fill analysis showed no missing studies, reinforcing the reliability of the association. This suggests that the TaqI heterozygous genotype significantly contributes to CSU risk.

###### BsmI polymorphism

**FIGURE 5:**
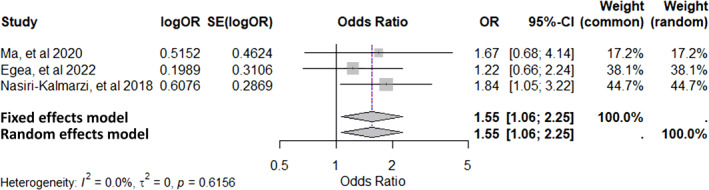
Forest plot summarizing the association between the BsmI vitamin‐D‐receptor polymorphism and chronic spontaneous urticaria (CSU). For each of the three included studies, the grey square marks the odds ratio (OR) and its horizontal bar the 95% confidence interval (CI), with square area proportional to the study’s inverse‐variance weight. The grey diamonds shows the fixed‐effect pooled estimate (OR = 1.55, 95% CI 1.06–2.25), and the random‐effects pooled estimate (OR = 1.55, 95% CI 1.06–2.25); the diamond tips correspond to the pooled CI. The solid vertical line at OR = 1.0 denotes “no association,” while the red dashed and blue dotted vertical lines mark the fixed‐ and random‐effects pooled ORs, respectively. Negligible between‐study heterogeneity was detected (Q = 0.83, *I*² = 0%, *τ*² = 0, *p* = 0.6156). So the fixed‐ and random‐effects estimates coincide and either can be interpreted as the overall effect size. The plot demonstrates a statistically significant increase in CSU susceptibility among carriers of the BsmI polymorphism.

The BsmI polymorphism (rs1544410) analysis included data from Ma et al.,^56^ Egea et al.,^63^ and Nasiri‐Kalmarzi et al.^48^ The pooled OR was 1.55 (95% CI: 1.06–2.25) under both models, with no heterogeneity (*I* ^2^ = 0.0%, *τ* ^2^ = 0, *p* = 0.6156) (Figure 5). The funnel plot showed symmetry indicating no publication bias. Individual study ORs were 1.67 (95% CI: 0.68–4.14) for Ma et al.,^56^ 1.22 (95% CI: 0.66–2.24) for Egea et al.,^63^ and 1.84 (95% CI: 1.05–3.22) for Nasiri‐Kalmarzi et al.,^48^ with respective study weights of 17.2%, 38.1%, and 44.7%. This confirms a significant association between the heterozygous genotype and CSU susceptibility.

#### 3.2.2 Risk of bias assessment of the HLA studies

The meta‐analysis of HLA‐B44 and its association with chronic urticaria included three studies: Bozek et al.,^31^ Coban et al.,^29^ and Pacor et al.^25^ All studies demonstrated a low RoB, scoring 8/9 on the NOS (Table S1). Each study provided adequate case definitions, representative patient populations, appropriately selected and clearly defined controls, and consistent genotyping methods (e.g., PCR‐SSP). However, none of the studies reported non‐response rates, which limits the ability to assess selection bias due to non‐participation. Notably, Coban et al.^29^ had a smaller sample size (40 patients and 30 controls), potentially reducing statistical power and limiting generalisability. Despite the uniformly high NOS scores, the pooled analysis revealed substantial between‐study heterogeneity (Q = 14.29, *I*² = 86 %, *τ*² = 1.6513, *p* = 0.0008), indicating that methodological or population differences not fully captured by the NOS—such as limited covariate adjustment, variations in allele frequency across ethnic groups, or unmeasured environmental interactions—may be influencing effect sizes. Only Bozek et al. adjusted for age and sex, leaving the possibility of residual confounding in the other two studies. In addition, quality‐control procedures for genotyping (duplicate assays, Hardy–Weinberg equilibrium testing in controls) were incompletely described by Pacor et al. and Coban et al. Taken together, these factors warrant cautious interpretation of the magnitude of the pooled random‐effects estimate, even though the overall direction of association remains robust.

#### 3.2.3 Risk of bias assessment of the VDR gene polymorphisms studies

The meta‐analysis of VDR gene polymorphisms (FokI, TaqI, and BsmI) included four studies: Ma et al.,^56^ Khoshkhui et al.,^60^ Egea et al.,^63^ and Nasiri‐Kalmarzi et al.^48^ All studies demonstrated a low RoB, each scoring 8/9 on the NOS (Table S2). These studies provided adequate case definitions, representative patient populations, clearly defined and appropriately selected controls, and consistently employed rigorous genotyping techniques such as PCR‐RFLP and TaqMan probes. Confounding factors, including age and gender, were addressed through matching or statistical adjustment. However, non‐response rates were not reported in any of the studies, and the region‐specific populations may limit generalisability to broader contexts.

Heterogeneity varied across the meta‐analyses of individual polymorphisms. For the FokI polymorphism, low heterogeneity was observed (*I* ^2^ = 0%), indicating consistent findings across studies. In contrast, the TaqI polymorphism showed moderate heterogeneity (e.g., *I* ^2^ = 34%), reflecting some variability in the effect estimates. For the BsmI polymorphism, low heterogeneity (*I* ^2^ = 0%) again highlighted agreement among studies. Despite these variations, the consistent methodologies and overall low RoB across the included studies reinforce the reliability of the pooled estimates, supporting significant associations for VDR polymorphisms in chronic urticaria.

## 4 DISCUSSION

This systematic review and meta‐analysis provide robust evidence highlighting the pivotal role of genetic factors in CU pathogenesis. Genetic polymorphisms, particularly within immune‐regulatory genes and associated pathways, significantly influence disease susceptibility, clinical severity, and therapeutic responsiveness. Notable associations, such as those involving HLA‐B44 and VDR polymorphisms, including TaqI and FokI, underscore the critical genetic determinants contributing to CU. These findings emphasise the centrality of immune modulation in CU pathophysiology and suggest that genetic predispositions may act in concert with environmental and epigenetic factors to facilitate disease manifestation.

The role of the immune system in CU is further supported by findings from GWAS and gene expression analyses. These studies have identified loci near MHC and ITPKB as well as key pathways such as JAK‐STAT, TNF, and NF‐κB, as being pivotal in CU pathogenesis. These findings not only reinforce the autoimmune component of CU but also reveal its genetic overlap with other immune‐mediated conditions. This genetic overlap provides a potential explanation for the observed increased prevalence of autoimmune conditions, such as thyroid disease, among CU patients.

From a therapeutic perspective, genetic insights present significant potential for advancing personalised medicine in CU management. Polymorphisms in genes such as CRTH2 and FcɛRI have been associated with variability in response to antihistamines, offering a pathway to optimise treatment strategies based on individual genetic profiles. Furthermore, emerging evidence from gene expression studies highlights the potential of targeting specific inflammatory pathways, such as those mediated by CCL2 and TNF, as a basis for developing innovative therapies to address refractory cases of CU.

Despite these advancements, significant gaps remain. The majority of studies have focussed on CSU, with limited exploration of other CU subtypes or AU. Moreover, variations in study design, sample size, and population demographics contribute to heterogeneity, complicating the synthesis and interpretation of findings. The lack of longitudinal studies to explore gene‐environment interactions further limits our understanding of the dynamic processes driving CU pathogenesis.

Future research should prioritise expanding genetic studies to include a wider range of CU subtypes and diverse populations, as well as investigating epigenetic modifications that may impact disease expression. Collaborative efforts across multiple centres and standardised methodologies will be essential to validate potential biomarkers and therapeutic targets. Incorporating genetic insights into clinical practice has the potential to transform CU management through the implementation of precision diagnostics and individualised treatment approaches.

This synthesis of current evidence highlights the complex genetic landscape of CU and highlights opportunities for further exploration into its pathophysiology and therapeutic strategies. By addressing existing gaps, we can move closer to developing tailored interventions that are expected to improve clinical outcomes for CU patients.

## 5 CONCLUSION

In conclusion, this systematic review and meta‐analysis emphasise the significant role of genetic factors in the pathogenesis, diagnosis, and treatment of CU. Key findings include the identification of significant associations between genetic polymorphisms—such as those in HLA‐B44 and VDR genes—and disease susceptibility, highlighting the potential for genetic biomarkers to improve diagnostic precision and guide personalised therapeutic strategies. The evidence further reinforces the immune‐mediated nature of CU, aligning its genetic background with that of other autoimmune conditions and classifying CU as one of the less harmful types of autoimmune disorders.^76^ Despite the progress, there is a need for comprehensive studies to address existing gaps, including the exploration of non‐CSU subtypes and AU, epigenetic modifications, and gene‐environment interactions. Integrating genetic findings into clinical practice is expected to advance precision diagnostics and personalised treatments, ultimately improving the quality of care for CU patients. Future research should focus on longitudinal studies and the exploration of gene‐environment interactions to gain a more comprehensive understanding of the dynamic nature of CU.

## AUTHOR CONTRIBUTIONS

**George N. Konstantinou**: Conceptualization, systematic review, meta‐analysis, writing. **Indrashis Podder**: Systematic review, writing—original draft. **Arunima Dhabal**: Systematic review, writing—original draft.

## CONFLICT OF INTEREST STATEMENT

The authors declare no conflicts of interest.

## Supporting information

Supporting Information S1

Supporting Information S2

Table S1

## Data Availability

Detailed data are available from the authors upon reasonable request.
