# Supplementary material for: Is there a role of genetics in acute and chronic urticaria—A systematic review and meta‐analysis
Source: Clin Transl Allergy. 2025 Jul 9;15(7):e70072. doi: 10.1002/clt2.70072 (PMC12240874; doi:10.1002/clt2.70072)
Supplement: Supplementary file 1 — Supporting Information S1 [file CLT2-15-e70072-s003.docx]

**RoB Tables**

**Supporting Table 2.** Detailed Risk Of Bias For HLA Studies

| Study | Case Definition Adequate | Representativeness of Cases | Selection of Controls | Definition of Controls | Control for Confounding Factors (Matching/Adjustment) | Ascertainment of Exposure | Same Method for Cases and Controls | Non-response Rate Similarity | Total Score | RoB Assessment |
| --- | --- | --- | --- | --- | --- | --- | --- | --- | --- | --- |
| Bozek et al. (2010) | 1 | 1 | 1 | 1 | 1 | 1 | 1 | 0 | 8 | Low |
| Coban et al. (2008) | 1 | 1 | 1 | 1 | 1 | 1 | 1 | 0 | 8 | Low |
| Pacor et al. (2006) | 1 | 1 | 1 | 1 | 1 | 1 | 1 | 0 | 8 | Low |

**Supporting Table 3.** Detailed Risk Of Bias For VDR Polymorphism Studies

| Study | Case Definition Adequate | Representativeness of Cases | Selection of Controls | Definition of Controls | Control for Confounding Factors (Matching/Adjustment) | Ascertainment of Exposure | Same Method for Cases and Controls | Non-response Rate Similarity | Total Score | RoB Assessment |
| --- | --- | --- | --- | --- | --- | --- | --- | --- | --- | --- |
| Ma et al. (2020) | 1 | 1 | 1 | 1 | 1 | 1 | 1 | 0 | 8 | Low |
| Khoshkhui et al. (2021) | 1 | 1 | 1 | 1 | 1 | 1 | 1 | 0 | 8 | Low |
| Egea et al. (2022) | 1 | 1 | 1 | 1 | 1 | 1 | 1 | 0 | 8 | Low |
| Nasiri-Kalmarzi et al. (2018) | 1 | 1 | 1 | 1 | 1 | 1 | 1 | 0 | 8 | Low |
