# Supplementary material for: Is there a role of genetics in acute and chronic urticaria—A systematic review and meta‐analysis
Source: Clin Transl Allergy. 2025 Jul 9;15(7):e70072. doi: 10.1002/clt2.70072 (PMC12240874; doi:10.1002/clt2.70072)
Supplement: Supplementary file 2 — Supporting Information S2 [file CLT2-15-e70072-s002.docx]

**Supporting Box 1:** Search Strategies

| **PubMed Search Strategy**  Search Terms:  (“genes*”[Title/Abstract] OR “genet*”[Title/Abstract] OR “genom*”[Title/Abstract] OR “polymorph*”[Title/Abstract]) AND “urticari*”[Title/Abstract] AND “humans”[MeSH Terms] AND “English”[Language]) AND (1000/1/1:2024/7/31[pdat]) |
| --- |
| **SCOPUS Search Strategy**  Search Terms:  (TITLE-ABS (urticaria*)) AND ((TITLE-ABS (genes*) OR TITLE-ABS (genet*) OR TITLE-ABS (genom*) OR TITLE-ABS (polymorph*))) AND (LIMIT-TO (SRCTYPE “j”)) AND (LIMIT-TO (DOCTYPE “ar”)) AND (LIMIT-TO (LANGUAGE “English”)) AND (LIMIT-TO (EXACTKEYWORD “Human”) OR LIMIT-TO (EXACTKEYWORD “Humans”)) |
| **Web of Science Search Strategy**  Search Terms:  (((AB=(genes*)) OR AB=(genet*)) OR AB=(genom*)) OR AB=(polymorph*)) AND AB=(urticari*) AND Document Types: Article AND Languages: English \| Timespan: 1900-01-01 to 2024-07-31 (Publication Date) |
