# Supplementary material for: Is there a role of genetics in acute and chronic urticaria—A systematic review and meta‐analysis
Source: Clin Transl Allergy. 2025 Jul 9;15(7):e70072. doi: 10.1002/clt2.70072 (PMC12240874; doi:10.1002/clt2.70072)
Supplement: Supplementary file 3 — Table S1 [file CLT2-15-e70072-s001.docx]

**Supporting Table 1:** Search Strategies

**PubMed Search Strategy**

Search Terms:
 - ("genes*"[Title/Abstract] OR "genet*"[Title/Abstract] OR "genom*"[Title/Abstract] OR "polymorph*"[Title/Abstract])
 AND "urticari*"[Title/Abstract] AND ("humans"[MeSH Terms] AND "english"[Language])
 AND (1000/1/1:2024/7/31[pdat])

**SCOPUS Search Strategy**

Search Terms:
 - (TITLE-ABS (urticaria*)) AND ((TITLE-ABS (genes*) OR TITLE-ABS (genet*) OR TITLE-ABS (genom*) OR TITLE-ABS (polymorph*)))
 AND (LIMIT-TO (SRCTYPE "j")) AND (LIMIT-TO (DOCTYPE "ar")) AND (LIMIT-TO (LANGUAGE "English"))
 AND (LIMIT-TO (EXACTKEYWORD "Human") OR LIMIT-TO (EXACTKEYWORD "Humans"))

**Web of Science Search Strategy**

Search Terms:
 - (((AB=(genes*)) OR AB=(genet*)) OR AB=(genom*)) OR AB=(polymorph*)) AND AB=(urticari*)
 AND Document Types: Article
 AND Languages: English
 | Timespan: 1900-01-01 to 2024-07-31 (Publication Date)
